# Targeting the NRF2/HO-1 Antioxidant Pathway in FLT3-ITD-Positive AML Enhances Therapy Efficacy

**DOI:** 10.3390/antiox11040717

**Published:** 2022-04-05

**Authors:** Sankaranarayan Kannan, Mary E. Irwin, Shelley M. Herbrich, Tiewei Cheng, LaNisha L. Patterson, Marisa J. L. Aitken, Kapil Bhalla, M. James You, Marina Konopleva, Patrick A. Zweidler-McKay, Joya Chandra

**Affiliations:** 1 Department of Pediatrics Research, The University of Texas M.D. Anderson Cancer Center, Houston, TX 77030, USA; mary.irwin.phd@gmail.com (M.E.I.); smherbrich@mdanderson.org (S.M.H.); tiewei.cheng@gmail.com (T.C.); lapatter@utmb.edu (L.L.P.); amarisa@med.umich.edu (M.J.L.A.); patrick.zweidler-mckay@immunogen.com (P.A.Z.-M.); 2 Leukemia, The University of Texas M.D. Anderson Cancer Center, Houston, TX 77030, USA; kbhalla@mdanderson.org (K.B.); mkonople@mdanderson.org (M.K.); 3 University of Texas MD Anderson UT Health Graduate School of Biomedical Sciences, Houston, TX 77004, USA; 4 Hematopathology, The University of Texas M.D. Anderson Cancer Center, Houston, TX 77030, USA; mjamesyou@mdanderson.org; 5 ImmunoGen, Waltham, MA 02451, USA; 6 Epigenetics and Molecular Carcinogenesis, The University of Texas M.D. Anderson Cancer Center, Houston, TX 77030, USA

**Keywords:** FLT3-ITD, TKI resistance, HO-1, NRF2, AML

## Abstract

Acute myeloid leukemia (AML) is a molecularly heterogenous hematological malignancy, with one of the most common mutations being internal tandem duplication (ITD) of the juxtamembrane domain of the fms-like tyrosine kinase receptor-3 (FLT3). Despite the development of FLT3-directed tyrosine kinase inhibitors (TKI), relapse and resistance are problematic, requiring improved strategies. In both patient samples and cell lines, FLT3-ITD raises levels of reactive oxygen species (ROS) and elicits an antioxidant response which is linked to chemoresistance broadly in AML. NF-E2–related factor 2 (NRF2) is a transcription factor regulating the antioxidant response including heme oxygenase -1 (HO-1), a heat shock protein implicated in AML resistance. Here, we demonstrate that HO-1 is elevated in FLT3-ITD-bearing cells compared to FLT3-wild type (WT). Transient knockdown or inhibitor-based suppression of HO-1 enhances vulnerability to the TKI, quizartinib, in both TKI-resistant and sensitive primary AML and cell line models. NRF2 suppression (genetically or pharmacologically using brusatol) results in decreased HO-1, suggesting that TKI-resistance is dependent on an active NRF2-driven pathway. In AML-patient derived xenograft (PDX) models, brusatol, in combination with daunorubicin, reduces leukemia burden and prolongs survival. Cumulatively, these data encourage further development of brusatol and NRF2 inhibition as components of combination therapy for refractory AML.

## 1. Introduction

Acute myelogenous leukemia (AML) afflicts ~20,830 new patients per year in the United States, with a current overall survival rate of merely 25.6% [1,2]. One of the most common mutations in AML is internal tandem duplication (ITD) of the juxtamembrane domain of the fms-like tyrosine kinase receptor-3 (FLT3) gene, which renders the encoded protein constitutively active [3]. This mutation is seen in approximately 30% of patients with AML [4] and expression correlates with poor clinical prognosis [4,5]. Consequently, multiple inhibitors have been generated that target the FLT3-receptor, with two tyrosine kinase inhibitors (TKI) currently being FDA-approved for AML: midostaurin and gilteritinib. Quizartinib is a second-generation TKI with increased potency and selectivity for FLT3 and has shown promise in terms of improved remission rates and overall survival in relapsed/refractory FLT3-mutated AML patients [6,7,8]. However, FLT3-directed TKI have shown only modest benefit as single agents in clinical trials [8], and relapse and resistance remain significant challenges for patients with FLT3-ITD^+^AML requiring the design of effective combination therapies [9].

In addition to promoting survival and growth signaling, oncogenic tyrosine kinases such as FLT3-ITD are capable of inducing elevated levels of reactive oxygen species (ROS) [10]. Indeed, in both patient samples and cell lines, the FLT3-ITD oncogene has been shown to promote the induction of ROS [11], which are thought to promote proliferative signaling and elicit an adaptive response to oxidative stress. Supporting this concept, many antioxidant proteins are increased in AML cells, including the small heat shock protein, heme oxygenase-1 (HO-1). In non-FLT3 mutant AML, HO-1 contributes to resistance to tumor necrosis factor (TNF)-induced apoptosis and to epigenetically targeted agents [12]. The transcription factor NRF2 is one of the major drivers of HO-1 expression and has also been linked to drug resistance in AML [13]. A natural product, brusatol, isolated from the seeds of Brucea javanica or sumatrana, has been found to inhibit NRF2 through a post-transcriptional mechanism, and can act as a chemosensitizer to several clinically relevant AML therapies including cytarabine, daunorubicin, and arsenic trioxide in AML cell lines and primary AML patient samples [14]. Despite documented anti-tumor activity in numerous models, the molecular target for brusatol is elusive; however, effects on NRF2 signaling have been cited extensively. None of these prior studies have focused on FLT3 mutant AML or evaluated the influence of brusatol on TKI efficacy in these settings. Here, we report that HO-1 and NRF2 are upregulated in FLT3 mutant AML and that genetic or pharmacological targeting of either of these targets enhances TKI efficacy. Our studies are extended to in vivo and AML primary patient samples and support the future development of combination therapy approaches composed of FLT3-directed TKI and the targeting of antioxidant pathways in AML. 

## 2. Materials and Methods

### 2.1. Cell Lines and Chemicals

Human AML cell lines (KG-1, THP-1, MOLM13, MOLM13-TKIR and MV411) and all BaF3 mouse lines were maintained in RPMI1640 with 10% FBS, 1% L-glutamine, and 1% penicillin/streptomycin. All cell lines were cultured in 5% CO_2_ at 37 °C. KG-1, THP-1, MOLM13 and MV411 cells were acquired from American Type Culture Collection (ATCC, Manassas, VA, USA). Cell lines were confirmed by Short Tandem Repeat (STR) DNA profiling protocol in MD Anderson Cancer Center Characterized Cell Line Core. All cell lines were free of mycoplasma contamination as confirmed by testing every 6 months. BaF3 FLT3 wildtype (WT) and TKI resistant FLT3 mutant cell lines (e.g., point mutants D835V and F691V) were generously provided by Dr. Donald Small at Johns Hopkins University. BaF3/FLT3-WT cells were additionally supplemented with 2 ng/mL recombinant mouse interleukin 3. To create BaF3/FLT3-ITDR cells, BaF3/FLT3-ITD cells were treated with increasing doses of lestaurtinib (LC Laboratories, Woburn, MA, USA) over a period of two to three months. ITDR cells were tested for resistance to FLT3-directed TKI and non-TKI as measured by dose responses and significant differences in IC_50_ values. ITDR cells were continually supplemented with 17.5 nM lestaurtinib until one week prior to the experiments shown. Quizartinib was purchased from LC Laboratories (Woburn, MA, USA). Brusatol was purchased from Ark Pharm (Cat. No. 128303; Arlington Heights, IL, USA). De-identified primary patient AML samples were collected with approval from the University of Texas MD Anderson IRB under LAB05-0511. 

### 2.2. Data Mining

Illumina HiSeq RNASeqV2 level 3, Illumina HiSeq DNASeq mutation, and clinical data for the TCGA AML cohort used for the survival analysis were downloaded from the TCGA data portal current as of 8 January 2014. Only the 173 patients with information on gene expression, mutation, and survival were used. Gene expression data were log2 transformed. To avoid errors for RNASeq raw counts of 0 all values are offset by 1 prior to taking logs. FLT3-ITD+ patients were classified as those with in-frame insertions in the FLT3 gene. The test for differences in survival was performed by comparing the overall survival of patients in the top top 66th and bottom 33rd percentiles percentiles of HO-1 gene expression using the log-rank test included in the ‘survival’ package in R. 

### 2.3. Detection of Protein

Cells were treated as indicated in text and figure legends and then lysed for 1 h using triton lysis buffer (PBS with 1% Triton X-100; 25 mM Tris, pH 7.5; and 150 mM NaCl) with protease inhibitor cocktail (Roche, Indianapolis, IN, USA) at 4 °C. Protein abundance was measured by Bradford assay (Bio-Rad, Hercules, CA, USA). Laemelli buffer was added to samples and the tube was boiled for 5 min, and then was subjected to SDS-PAGE and transferred to PVDF membrane (Bio-Rad) [15]. Membranes were blocked as per the manufacturer’s instructions then exposed to HO-1 (Cat No. ADI-SPA-895, Enzo, Farminglade, NY, USA), NRF2 (SC-722, Santa Cruz Bio, Dallas, TX, USA), or Actin (Cat. No. A2228, Millipore Sigma, Burlington, MA, USA) antibodies overnight. Membranes were washed with tris buffered saline containing 1% Tween-20 (TBST) for 30 min followed by incubation with secondary antibodies (GE Healthcare, Pittsburgh, PA) [15]. Visualization was performed using ECL (Bio-Rad) and densitometry measured using NIH ImageJ (Bethesda, MA, USA).

### 2.4. Real-Time PCR

RNeasy Mini Kit (QIAGEN, Valencia, CA, USA) was used to purify RNA followed by reverse transcription reaction (1 μg of RNA) using iScript RT kit (Bio-Rad). Real-time PCR using the iTaq Universal SYBR Green PCR master mix (Bio-Rad) in a 20 µL total volume was performed. The PCR primer sequences and conditions for HO-1, GPX, and NRF2 were previously described [16]. Relative gene expression was calculated by determining the cycle threshold (C_t_) value and normalizing to Actin.

### 2.5. Knockdown Experiments

HO-1 siRNA (purchased from Santa Cruz Biotechnology, Dallas, TX, USA, Cat# sc-35555) was transfected into all cell lines using lipofectamine RNAiMAX (Life Technologies, Waltham, MA, USA), and Nucleofector Kit (Lonza, Basel, Switzerland) according to the manufacturer’s instructions. shRNA Nrf2 (Cat#: TG311194) and OriGene retroviral particles were transduced using protocol published earlier [17]. Knockdown was evaluated by Western blotting as described earlier. Twenty-four hours after knockdown, cells used for further experiments were treated with indicated drugs for 48 h unless otherwise indicated.

### 2.6. Proliferative/Cell Death Analysis

Cells were treated as indicated and then harvested. Proliferation was assessed by cell counting via hemocytometer (Thermo-Fisher, Waltham, MA, USA) with trypan blue (Thermo-Fisher) or Alamar blue (Thermo-Fisher) staining to indicate viability. Alternatively, cells were harvested and counted using a ViCell analyzer (Beckman-Coulter, Indianapolis, IN, USA). Cell cycle was analyzed utilizing annexin-V/propidium iodide (Thermo-Fisher) staining followed by flow cytometry on a BD FACScalibur flow cytometer. Percent sub-diploid was then measured using CellQuant software as described by Kannan et al., 2019 [17].

### 2.7. Assessment of the Redox Environment

ROS were measured by staining with dichlorofluorescein (DCF) followed by flow cytometry. Briefly, equal cell numbers were stained with DCF (Life Technologies, Waltham, MA, USA) as per the manufacturer’s instructions. Samples were analyzed on the FL-1 channel of a flow cytometer. The mean fluorescence of 10,000 cells was then normalized to controls. The redox state of GSH (GSH/GSSG) was determined in cell extracts as described previously [16,18]. MitoSOX red (Cat. M36008, Thermo Fisher) reagent was used for mitochondrial superoxide detection as per the manufacturer’s instructions.

### 2.8. Mouse Studies

All in vivo studies were performed with approval from the University of Texas MD Anderson Institutional Animal Care and Use Committee (IACUC). NOD-SCID/IL2Rg-immunodeficient mice constitutively expressing three human myeloid cytokines, (IL-3, SCF and GM-CSF; NSGS, Jax Labs, Stock 013062) were injected through the tail vein with 0.5 × 10^6^ MOLM13 or MOLM13-TKIR cells as described previously [17,19]. PDX models were established from primary AML cells as previously described by tail-vein injection [14,16]. For PDX models, engraftment was confirmed by a leukemic burden in the peripheral blood of 0.5–2% as measured by human CD45 staining and flow cytometry. This level of peripheral blood blasts reflects bone marrow blasts of approximately 20–30%, which mimics human AML diagnostic criteria. After engraftment, leukemia-bearing animals were injected i.v. (intravenousely through the tail vein) with 2 mg/kg brusatol (in PBS) or vehicle (PBS) every other day for a total of five injections. Animals were bled weekly for the monitoring of leukemia burden. Briefly, red blood cells were lysed with ACK lysis buffer (Cat No A1049201, Thermo-Fisher) then washed with PBS. After Fc-block, cells were stained with FITC-anti-CD45 antibody and read on the FITC channel of a BD Fortessa flow cytometer. Animals were monitored daily for moribund features such as hind-limb paralysis and sacrificed accordingly. Survival curves were created using GraphPad Prism, and statistical analyses were performed [17]. 

### 2.9. Statistical Analyses

Unless otherwise stated, values listed in figures are expressed as the mean ± standard error of the mean (SEM) of at least three replicates. Statistical comparisons were made using GraphPad Prism 4.0 software (GraphPad Software, Inc., La Jolla, CA, USA) by means of the Mantel–Cox test (survival analyses) or Student’s t-test. A *p*-value of * < 0.05 was considered significant. IC_50_ calculations were made using GraphPad Prism 4.0. Combination index values were calculated using the computer software program Calcusyn. A combination index value of less than 1 indicated synergy, a value equal to 1 indicated additivity, and a value greater than 1 indicated antagonism. Experiments were repeated at least three times. 

## 3. Results

### 3.1. HO-1 Promotes Proliferation and Survival in FLT3-ITD Expressing AML

**Figure 1:**
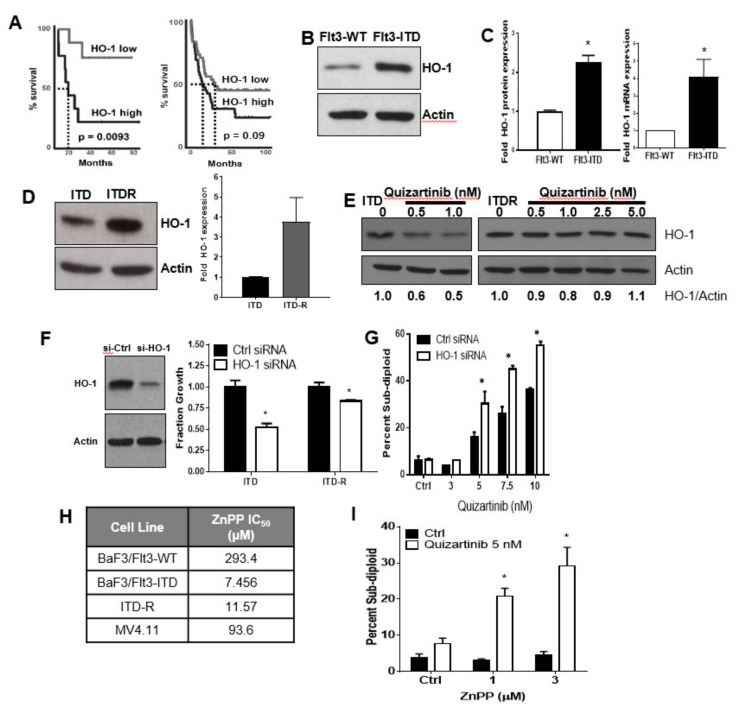
HO-1 promotes proliferation and survival in FLT3-ITD expressing AML. (**A**) Kaplan–Meier survival curves show a significant increase for the overall survival of the low HO-1 gene-expressing patient population (*n* = 9) compared to the high HO-1-expressing Flt3-ITD-positive AML patients (*n* = 9) (left panel) versus no significant difference in non-FLT3-ITD mutant AML patients (right panel) in high (*n* = 42) versus low (*n* = 41) HO-1 expression (TCGA data). (**B**) FLT3-WT or FLT3-ITD) cell lysates (50 µg/lane) were loaded and the Western blot was performed for HO-1 and actin. (**C**) HO-1 mRNA gene expression in FLT3-WT and FLT3-ITD mutant-expressing BaF3 cells are shown by q-RTPCR assay. (**D**) FLT3-ITD mutant BaF3 cells (ITD) and a novel model of acquired pan-FLT3-inhibitor resistance BaF3-ITD cells (ITD-R) were used for protein lysate preparation, and Western blot analysis (50 µg/lane) for HO-1 and actin protein levels was performed with densitometry shown adjacent. (**E**) HO-1 protein expression in BaF3-ITD and -ITD-R cells treated with a second-generation FLT3 kinase inhibitor at the indicated concentration were used for protein lysate preparation and te detection of HO-1 and actin protein (50 µg/lane) levels by Western blot analysis. (**F**) Parental BaF3-ITD and ITD-R cells were transfected with si-HO-1 and scramble control si-RNAs after 48 h. Cell lysates were loaded on SDS-PAGE for the detection of HO-1 and actin by Western blot analysis ((**F**) left panel). Cell proliferation assay from HO-1 knock down cells shown on the (**F**) right panel. (**G**) HO-1 knocked down cells (ITD-R) were treated with quizartinib at the indicated concentration for 48 h. Stained with propidium iodide for the detection of percent of sub-diploid cells measured by flow cytometry. (**H**) A competitive inhibitor of HO-1, ZnPP, was directly used for the treatment of FLT3-WT, -ITD, and ITDR cells. The mean IC_50_ values found for three experiments are given in the table. (**I**) Bar diagram shows percent sub-diploid cells from the ratio of the mean DNA content of FLT3-ITDR cells treated with a combination of ZnPP and quizartinib at the indicated concentrations were measured by flow cytometry. Statistical significance is shown as *p*-values less than 0.05 (* *p* < 0.05). Each experiment was performed at least three times.

In order to adapt to increased levels of ROS caused by constitutive kinase activity of FLT3-ITD, antioxidants are often up-regulated [20]. One such antioxidant, HO-1, has unique functionality in that it can promote proliferative signaling resulting in survival and drug resistance in some cases [21]. To determine if HO-1 is altered in FLT3-ITD positive AML, we first mined data from The Cancer Genome Atlas (TCGA). We isolated the ITD-positive population and then stratified HO-1 expression into two categories: high (top 66%-expressing) or low (bottom 33%-expressing). Patients that expressed relatively high levels of HO-1 had decreased (*p* = 0.0093) survival as compared to those with low expression of HO-1 (Figure 1A) with nine patients in each group. This separation in survival curves was present in the FLT3-ITD-expressing population (left panel, Figure 1A), but not in patients without the FLT3-ITD mutation (right panel, Figure 1B).

**Figure 2:**
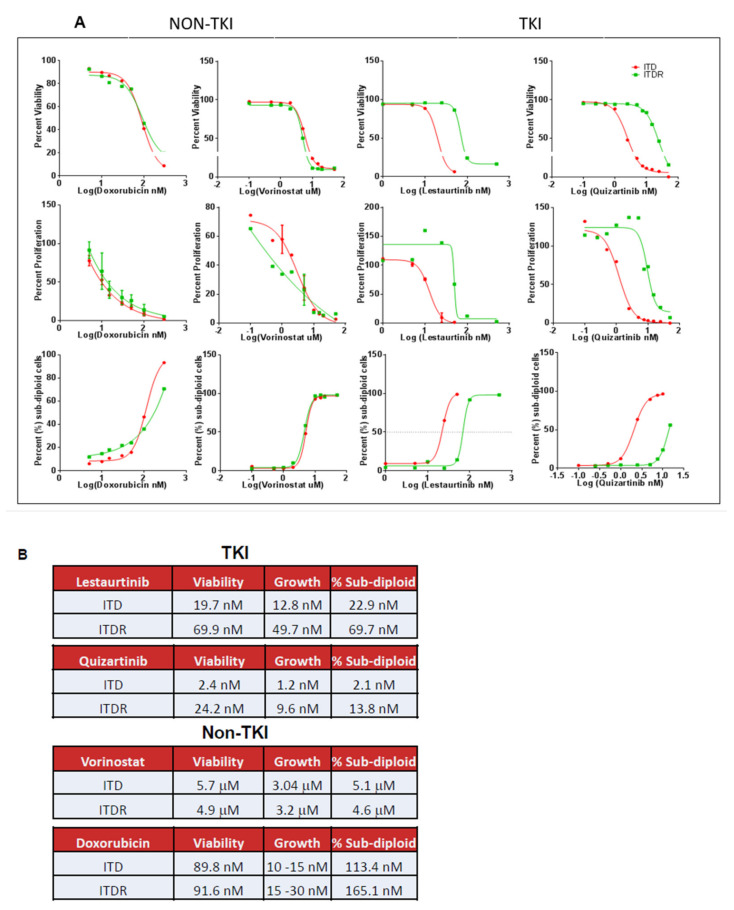
BaF3-FLT3-ITDR cells are pan-FLT3-inhibitor-resistant. (**A**) BaF3-FLT3-ITD and BaF3-FLT3-ITDR cells were treated with increasing doses of the FLT3-inhibitors lestaurtinib and quizartinib as well as the non-FLT3 inhibitors doxorubicin and vorinostat for 48 h. Proliferation and viability were measured using trypan blue exclusion and counting via ViCell analyzer. Cells were also stained with propidium iodide and measured on the FL-3 channel of a FACScaliber flow cytometer. (**B**) IC_50_ values were calculated based on viability, growth, and sub-diploid (PI) analyses using GraphPad Prism for BaF3-FLT3-ITD and BaF3-FLT3-ITDR cells.

To model this system in vitro, we utilized the isogenic murine cell line model of BaF3 transfected with either wild-type FLT3 (FLT3-WT) or FLT3 with the ITD mutation (FLT3-ITD) [22]. Western blotting of lysates from these cell lines indicated that HO-1 protein was significantly up-regulated in FLT3-ITD-positive cells as compared to FLT3-WT (Figure 1B). Furthermore, HO-1 mRNA expression in FLT3-ITD-expressing cells was also significantly increased as compared to wild-type (Figure 1C). As HO-1 has been noted to drive proliferative signaling resulting in resistance to chemotherapeutic agents in myelodysplastic syndrome [23], we used an isogenic pair of TKI-sensitive (ITD) and -resistant (ITDR) FLT3-ITD-expressing BaF3 cells to determine if HO-1 was also basally altered in TKI-resistant AML populations. Here, we developed a novel model of acquired pan-FLT3-inhibitor resistance in the isogenic BaF3 model cell line (FLT3-ITDR, Figure 2A,B). In the BaF3 TKI-sensitive and -resistant paired lines, HO-1 protein expression was significantly up-regulated in resistant cells as compared to their sensitive counterparts (Figure 1D). Together, these data suggest that FLT3-ITD-cohorts have elevated levels of the antioxidant HO-1, which may be further increased in TKI-resistant cell populations. 

Constitutive activation of the FLT3-receptor (as occurs with FLT3 mutations) initiates downstream signaling resulting in phosphorylation of transcription factors, which may result in increased expression of antioxidants. To determine if HO-1 protein expression was dependent on FLT3 kinase activity in our cell line models, we treated cells with increasing doses of the FLT3-directed TKI quizartinib. In parental FLT3-ITD-positive cells, treatment with low doses of quizartinib resulted in a 40–50% decrease in HO-1 protein (Figure 1E). However, in TKI-resistant cells, HO-1 expression was no longer dependent on FLT3 kinase activity (Figure 1E). Therefore, direct inhibition of HO-1, or the pathway controlling its expression, may be necessary in the setting of kinase inhibitor-resistant disease.

HO-1 has been noted to regulate proliferation and survival of a number of different cell types, including FLT3-WT AML [24,25]. Thus, to determine if targeting HO-1 may be relevant in the FLT3-ITD setting, we performed siRNA mediated knockdown of HO-1 protein (Figure 1F). Western blotting confirmed a successful knockdown of HO-1 in ITDR cells (Figure 1F, left panel). With knockdown, we noted a 50% decrease in the proliferation of parental ITD-expressing BaF3 cells and a 25% reduction in the proliferation of ITDR cells, suggesting that HO-1 targeting can modulate the proliferation of these cell lines (Figure 1F right panel). To determine if targeting HO-1 may be relevant to the sensitization of resistant cells to FLT3-directed TKI, we performed knockdown of HO-1 followed by treatment with quizartinib. After 48 h of treatment, cells were stained with propidium iodide and the percentage of sub-diploid cells was monitored by flow cytometry (Figure 1G). While quizartinib alone had little effect on ITDR cells at the doses tested, knockdown of HO-1 resulted in an approximately 2-fold increase in the sub-diploid population of cells in combination with quizartinib. To determine if the activity of HO-1 was required for this survival effect, we utilized a chemical means to inhibit HO-1 activity, namely zinc protoporphyrin (ZnPP) [26]. ZnPP treatment alone was more specific for ITD-positive cells compared to wild-type (Figure 1H). Much like the knockdown of HO-1, treatment of ITDR cells with the combination of ZnPP and quizartinib resulted in a synergistic two- to three-fold increase in apoptotic cells as compared to either drug alone (Figure 1I).

### 3.2. NRF2 Inhibition Decreases HO-1 Expression and Inhibits Viability in AML

**Figure 3:**
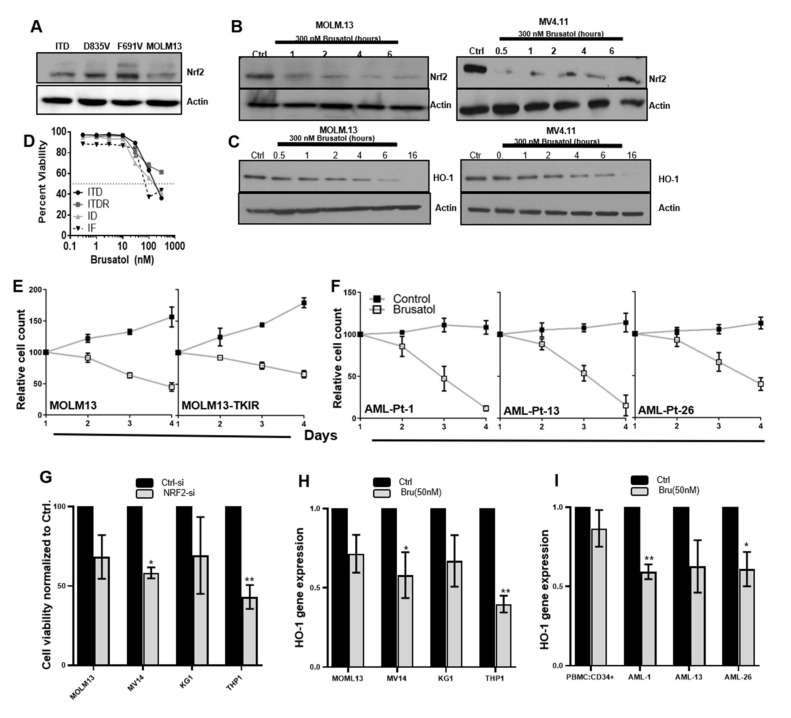
NRF2 inhibition decreases HO-1 expression and inhibits viability in AML. (**A**) Western blot analysis was performed on a panel of cell lines expressing FLT3-ITD (ITD, MOLM13) or FLT3-ITD mutants (D835V, -F691V, and MOLM13) to determine NRF2 and actin protein expression (**B**,**C**). MOLM13 and MV4.11 human ITD-expressing cell lines were treated with brusatol and cells were harvested at different time points for lysate preparation and Western blot analysis of NRF2 (**B**), HO-1 (**C**) and actin (both) protein expression was determined at indicated timepoints. (**D**) TKI-sensitive Ba/F3-Flt3-ITD cells and -resistant ITD cells (-ITDR, -D835V, -F691V) were treated with brusatol at indicated concentrations for 72 h, and then cell growth was analyzed. The brusatol concentration that inhibited 50% of cell viability (IC_50_) was determined from the dose–response curves. (**E**) MOLM13-sensitive and MOLM13-TKIR (tyrosine kinase inhibitor resistant) cells and (**F**) three patients’ primary AML samples were treated with NRF2 inhibitor brusatol (10 nM for 4-days) and vehicle, samples were stained with Alamar blue, and cell proliferation was measured quantitatively. (**G**) Cell viability assay was performed in a panel of AML cell lines after NRF2-si transfection in vitro. Transient knockdown of NRF2 by siRNA or control siRNA was allowed for 48 h, and cell viability was measured using ViCell counter. (**H**) HO-1 gene expression was measured from RNA lysates harvested from CD34+ve normal PBMC, and patients’ primary AML samples treated with 50 nM of brusatol for 24 h were harvested. HO-1 transcripts levels were measured by qRT-PCR. (**I**) HO-1 gene expression was measured from RNA lysates harvested from a panel of AML cell lines treated with 50 nM of brusatol for 24 h were harvested. HO-1 transcripts levels were measured by qRT-PCR. Statistical significance is shown as *p*-values less than 0.05 and 0.01 (* *p* < 0.05; ** *p* < 0.01). Experiments were repeated at least three times.

The master antioxidant transcription factor NRF2 is known to promote HO-1 expression in a variety of cellular systems [27]. We evaluated NRF2 protein expression in human and murine FLT3 mutant cells and found similar levels across cell types (Figure 3A). The NRF2 inhibitor, brusatol, is a natural product which exerts its effects by transiently decreasing NRF2 protein expression [28]. Therefore, to determine whether brusatol had the intended consequences on its target, NRF2, we treated MOLM13 and MV4.11 human ITD-expressing cell lines with brusatol over time (Figure 3B,C) using a dose representing the IC_50_ (Figure 3D) and then performed immunoblotting for NRF2 and the downstream target HO-1. As expected, both cell lines showed decreased protein expression of NRF2 and HO-1. Inhibition of HO-1 protein came at a later time point as compared to NRF2 (Figure 3C 16 h vs. 6 h), suggesting that HO-1 is downstream of NRF2. We next determined the effect on viability of treatment of both TKI-sensitive and TKI-resistant cell lines with brusatol. First, BaF3 isogenic cell line models were used that were TKI-sensitive (ITD), had acquired resistance (ITDR), or known point mutations of ITD that result in TKI resistance (ID). All ITD-expressing isogenic cell lines had similar dose–response graphs and IC_50_ values (Figure 3D), suggesting that inhibition of this protein is achievable in both populations. Next, the human ITD-expressing, TKI-sensitive cell line MOLM13 and its TKI-resistant counterpart (MOLM13-TKIR) were treated with brusatol for 4 days (Figure 3E). Both TKI-sensitive and -resistant human cell lines showed similar sensitivity to brusatol. Finally, three AML patient samples were treated over four days with brusatol (10 nM) and found to have similar sensitivity profiles to that of the human MOLM13 cells (Figure 3F). To confirm that these effects are specific to inhibition of NRF2, we determined the effects of NRF2 siRNA on cell viability in a panel of AML cell lines. Indeed, NRF2 downregulation through siRNA resulted in decreased cell proliferation of both FLT3-WT (KG1 and THP1) and -ITD (MOLM13 and MV4.11) expressing human cell lines in vitro (Figure 3G). As NRF2 is a known transcriptional regulator of HO-1, we treated FLT3-WT and -ITD expressing human cell lines and patient samples with brusatol to determine if this pathway was indeed intact in these cells (Figure 3H,I). Brusatol treatment resulted in decreased HO-1 expression in all cell lines tested. While brusatol has no effect on HO-1 expression in CD34+ PBMCs, treatment of three AML patient samples with brusatol resulted in a significant reduction in HO-1 gene expression. Together, these data suggest a pathway through which NRF2 disruption can result in decreased HO-1, resulting in decreased viability of AML cells without perturbing normal stem cells. 

### 3.3. Targeted NRF2 Inhibition Abrogates Reductive Stress and Promotes Anti-Leukemic Effects in AML

**Figure 4:**
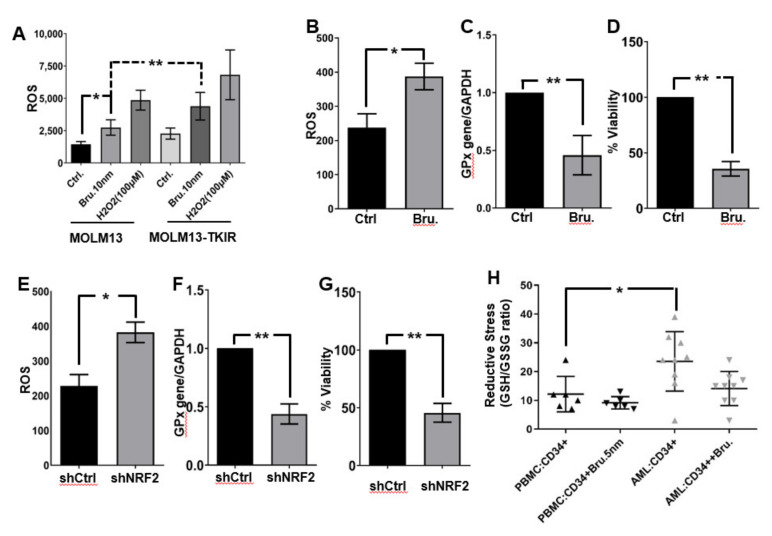
Targeted NRF2 inhibition promotes anti-leukemic effects in AML in vitro and ex vivo. (**A**). Flow cytometry detection of ROS in MOLM13 and MOLM-TKIR cells treated with brusatol and H_2_O_2_ for 24 h using DCF. (**B**). ROS levels were measured after brusatol treatment at 10 nM in patients’ primary AML samples (after 24 h) using DCF. (**C**). qRT-PCR based GPx gene expression levels were detected in samples from panel B. (**D**). Cell viability measured after 36 h in brusatol cells from panel B. (**E**–**G**). Same parameters as in (**B**–**D**), respectively, after NRF2 knock-down cells. (**H**). Ex vivo analysis of CD34^+^ cells from normal PBMC and patients’ primary AML cells, treated with brusatol for 24 h, and reduced/oxidized glutathione was measured as an indicator of reductive stress in AML. Biologically independent samples were plated in triplicates (as technical replicates), but statistics were performed only on biologically independent samples (*n* = 5), except for panel A and B. All experiments were repeated three times. Statistical significance is shown by *p*-values less than 0.05 and 0.01 (* *p* < 0.05; ** *p* < 0.01).

NRF2 is a transcriptional regulator of numerous cellular antioxidants, namely those that contain antioxidant response elements (ARE) in their promoters [29], and therefore its expression or inhibition is expected to influence cellular redox status. To determine the impact of NRF2 inhibition on the redox microenvironment in FLT3 mutant AML cells, we treated MOLM13 and MOLM13-TKIR cells with brusatol and then measured intracellular peroxide levels using DCF-DA staining (Figure 4A). Brusatol increased the levels of ROS in both TKI-sensitive and -resistant cell lines. To determine if this effect was also present in primary AML cells, we treated primary AML samples with brusatol for 24 h, and then again used DCF staining (Figure 4B). We also performed qRT-PCR for glutathione peroxidase (GPx; Figure 4C), an independent downstream target of NRF2, and determined cell viability using the Alamar blue assay (Figure 4D). ROS levels were significantly elevated by brusatol after 24 h of treatment. Concordantly, GPx gene expression was decreased. As noted earlier (Figure 3F), treatment of primary AML cells with brusatol decreased cell viability (Figure 4D) after 36 h. To confirm that these effects were directly related to inhibition of NRF2, we performed shRNA knockdown of NRF2 and measured these same endpoints (Figure 4E–G). Similar to inhibition with brusatol, shRNA-mediated knockdown of NRF2 resulted in increased levels of ROS (Figure 4E), decreased GPx expression (Figure 4F), and decreased cell viability (Figure 4G). Chronic ROS and oxidative stress are associated with carcinogenesis and mediate sustained NRF2 activation and antioxidants, leading to reductive stress [16]. Reductive stress is induced by excessive levels of reduced NAD^+^ (NADH), reduced NADP^+^ (NADPH), and GSH, and is implicated in many pathological process [30]. Cellular GSH levels are significantly (*p* * < 0.05) higher in CD34^+^ AML cells compared to normal peripheral blood mononuclear cells (PBMCs; Figure 4H). However, brusatol-exposed CD34^+^ AML cells showed comparable reduced GSH levels and redox (GSH/GSSG) ratio when compared with normal PBMCs, suggesting that the increased reducing power is diminished upon brusatol exposure (Figure 4H). Thus CD34^+^ AML cells demonstrated increased reductive metabolites (i.e., reductive stress) compared to normal PBMCs and brusatol treatment decreased aberrant reductive metabolite levels to baseline (GSH/GSSG ratio). 

### 3.4. Brusatol Potentiates Quizartinib Mediated Enhanced Anti-Leukemic Effect in AML

**Figure 5:**
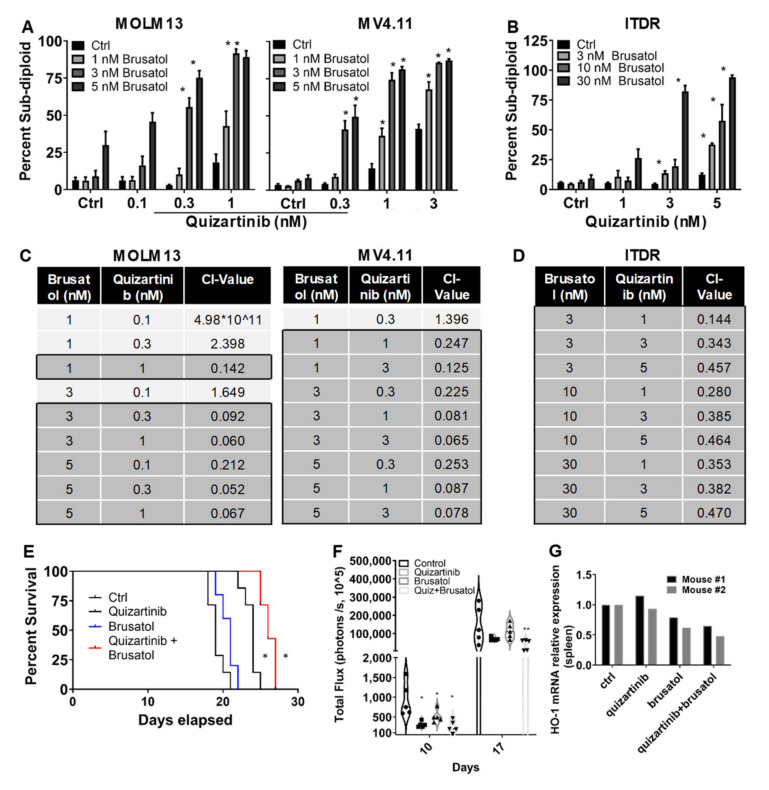
Brusatol potentiates quizartinib’s anti-leukemic effects in AML. (**A**) Bar diagram shows percentage of sub-diploid cells from the ratio of the mean DNA content of MOLM13 and MV4.11 human ITD-expressing cell lines and, (**B**) Ba/F3-Flt3-ITDR cell lines were treated with brusatol at the indicated concentrations and combined with quizartinib at the specified concentrations for 48 h. (**C**) Combination index values for combinations of the NRF2 inhibitor, brusatol, and FLT3 inhibitor, quizartinib, were measured in FLT3-ITD AML cell lines, MOLM13 and MV4.11. Synergy was determined based on DNA fragmentation assays using CalcuSyn software (Biosoft, Cambridge, United Kingdom), with combination index (CI) values < 1 considered synergistic, equal to 1 additive, and greater than 1 antagonistic. (**D**) Combination index values were obtained for the Ba/F3 Flt3-ITD-resitant cells treated with an inhibitor of NRF2 and an FLT3 inhibitor, quizartinib. (**E**) Prolonged survival upon combination of brusatol and quaizartinib in AML: overall survival of AML cell line xenograft-0derived AML-CDX. Briefly, 0.5 × 10^6^ MOLM13 cells in 200 µL of PBS were injected via the lateral tail vein in 250 Gy irradiated 5–6-week-old NSG-SGM3 mice (*n* = 7 per group). Engraftment of cells were monitored on a weekly basis through tail vein bleeding and staining of MOLM13 cells using human CD45 (hCD45) vs. mouse CD45 (mCD45) markers. When peripheral leukemic burden started showing, i.e., 0.5 to 2% treatment was started, Brusatol 2 mg/kg, 3 times per week, i.v.; quizartinib 10 mg/kg, 3 times per week, p.o. treatment was started. Each treatment was ended when individual mice reach moribund and survival data were recorded for interpretation. (**F**) In vivo efficacy of quizartinib (AC220), a uniquely potent and selective inhibitor of FLT3 and brusatol, an NRF2 inhibitor for the treatment of AML-PDX measured by bioluminescence imaging (BLI). Treatment schedules and doses of drug administered are mentioned in (**E**). Violin plots illustrate the individual whole mouse per each group responding to treatments over time. BLI represents bioluminescence intensity for each treatment group (p/s, 10^5^). (**G**) Human HO-1 gene expression was measured in mRNA extracts from mouse spleen treated with quizartinib or brusatol or combination of quizartinib and brusatol. Statistical significance is shown as *p*-values < 0.05 and 0.01 (* *p* < 0.05; ** *p* < 0.01).

In Figure 1G,I, we show that HO-1 inhibition using either knockdown or ZnPP leads to increased sensitivity to quizartinib in TKI-resistant cells. We hypothesized that targeting NRF2 with brusatol would show a similar phenotype in TKI-sensitive and -resistant cells. Indeed, when brusatol was combined with sub-IC_50_ doses of quizartinib in TKI-sensitive MOLM13 cell lines, there was a substantial increase in the percentage of sub-diploid cells compared to either drug alone (Figure 5A left panel). The same was true in the human FLT3-ITD expressing MV4.11 cell line (Figure 5A right panel). Additionally, when TKI-resistant BaF3-ITDR cells were treated with the combination of brusatol with quizartinib, there were similar increases in cell death, suggesting that brusatol was sufficient to sensitize the cells to FLT3-directed TKI (Figure 5B). The majority of combinations tested in all three cell lines were synergistic (Figure 5C,D), as calculated by combination index (CI) values below 1, suggesting a large therapeutic window for this combination. Next, to determine whether quizartinib-mediated inhibition of FLT3-ITD is associated with suppression of HO-1 gene expression through the targeting of NRF2, we conducted an in vivo study. MOLM13 cells were engrafted in NSG-SGM3 mice and treated with either quizartinib or brusatol alone and in combination. As observed in Figure 5E, single-arm treatment with either quizartinib or brusatol revealed increased survival with a significance of *p* < 0.05 when compared to vehicle-treated groups. Importantly, combination treatment resulted in a significant (*p* < 0.01) prolongation of survival as compared to the control or either drug alone. Additionally, the levels of HO-1 gene expression were measured in spleens harvested from the experimental groups used in Figure 5E. Quantification of bioluminescence values shows a significantly lowered tumor burden after brusatol and quizartinib treatment (Figure 5F). Brusatol treatment resulted in decreased HO-1 mRNA expression, while no change in HO-1 gene expression was seen in quizartinib-treated groups (Figure 5G). These data indicate that the NRF2 inhibitor, brusatol, was effective at reducing expression of HO-1 and enhanced anti-AML effects of FLT3-directed TKI in vivo.

### 3.5. Antioxidant Inhibition Promotes ROS-Spiking and Anti-Leukemic Effects in AML

**Figure 6:**
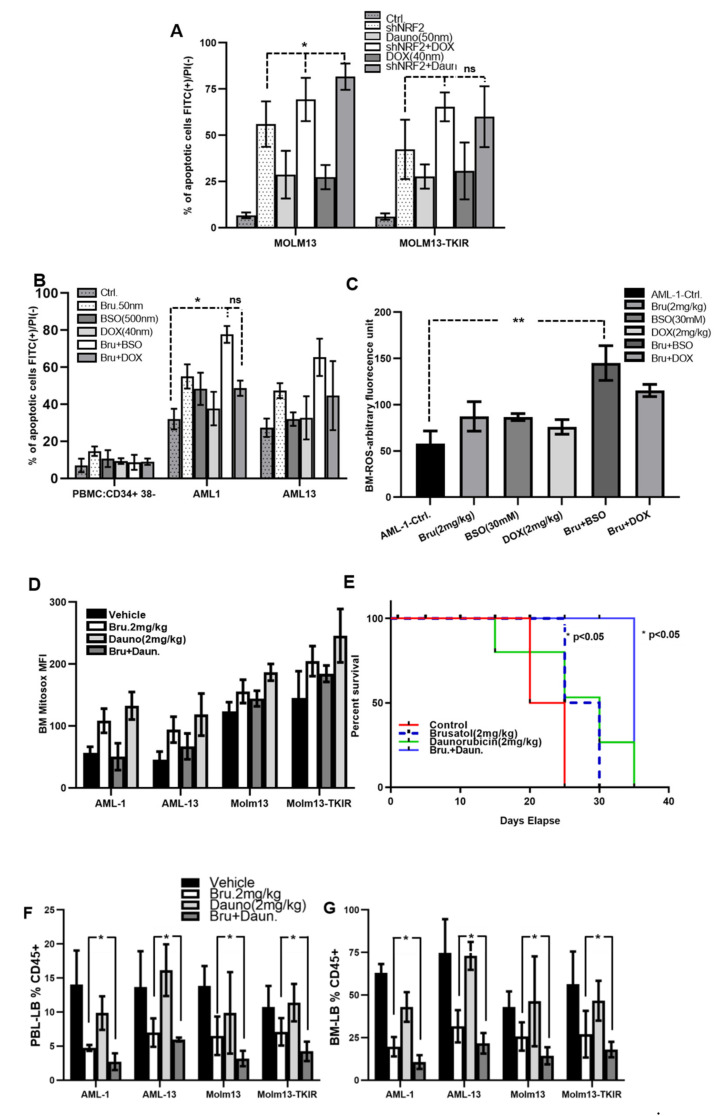
Pharmacologic inhibition of NRF2 potentiates chemotherapy mediated anti-leukemia activity in AML. (**A**) MOLM13 and MOLM13-TKIR cells were used for shNRF2 knockdown, treated with standard chemotherapy combination for 48 h in vitro, annexin-V FITC with PI staining was used for flow cytometry analysis. (**B**) Normal PBMC and primary AML samples treated with brusatol, BSO, DOX and their combinations for 48 h, annexin-V FITC with PI staining was used for flow cytometry analysis ex vivo. (**C**) ROS (total-cellular) levels in BM from AML-1-PDX was measured after targeted antioxidant inhibition using BSO, and brusatol, and chemotherapy treatment using DCF based flow cytometry ex vivo. (**D**) In vitro analysis showing mean fluorescence intensity (MFI) of mitochondrial-ROS levels in BM from AML-PDX, and MOLM13, and MOLM13-TKIR xenografted models are shown. Study cohorts treated with targeted antioxidant inhibition (brusatol) and chemotherapy (daunorubicin) were used for BM-Mitosox (5 µM/30 min incubation at 37 °C) staining, and fluorescence measured via flow cytometry. (**E**) Kaplan–Meier survival analysis from AML-PDX cohorts. (**F**) NSG-SGM3 mice engrafted with primary AML patients cells (AML-1, AML-13; 2 × 10^6^ cells were injected and mice with 2–5% of leukemic cells in peripheral blood (reflecting 20–30% tumor burden in bone marrow, observed in the third from initial injection) were treated, beginning 2 weeks after initial tail vein injection, with vehicle (control), brusatol (2 mg/kg), daunorubicin (2 mg/kg) by gavage thrice per week on alternative days for 2 weeks (*n* = 10 mice per group) and continuously monitored for 2 additional weeks. Mice were euthanized between weeks 4 and 5 due to features of moribundity such as hunched posture or hind leg paralysis after beginning treatment. (**F**-**G**) Leukemia burden was measured in the peripheral blood (**F**) or bone marrow (**G**) by hCD45 staining. Note: Biologically independent samples were plated in triplicates (as a technical replicates) but statistics were performed only on biologically independent samples, except panel A, B, and C). Statistical significance is shown by *p*-values less than 0.05 and 0.01 (* *p* < 0.05; ** *p* < 0.01) for the study arms or groups compared.

Standard of care for patients with AML includes treatment with anthracyclines (e.g., daunorubicin or doxorubicin) [2]. Interestingly, anthracyclines are capable of modulating the redox environment of cells [31]. Furthermore, in FLT3-WT AML, HO-1 is induced and promotes resistance to daunorubicin [4]. Therefore, to determine if there is a combined effect of NRF2/HO-1 pathway inhibition with anthracyclines, we treated MOLM13 and MOLM13-TKI cells with daunorubicin or doxorubicin alone, or in combination with shRNA directed against NRF2. After treatment, cells were stained with annexin-V/PI and analyzed by flow cytometry. Daunorubicin or doxorubicin alone showed moderate effects in either cell line at the doses utilized (50 nM and 40 nM, respectively). However, knockdown of NRF2 in these lines potently caused cell death (Figure 6A). Because the NRF2 knockdown was so effective, there was no significant additive increase in cell death as a result of combination treatment with NRF2 knockdown and anthracycline treatment. We further probed pharmacological approaches for inhibiting NRF2 targets. Since NRF2 is able to regulate glutathione (GSH) production, and elevated GSH is associated drug resistance [32] in AML cells, we used an inhibitor of GSH called buthionine sulfoxamine (BSO) along with brusatol and/or doxorubicin alone or in combination treatments on AML patients samples ex vivo. The results from BSO with brusatol combination show significant cell death compared to BSO and doxorubicin (Figure 6B). In addition, cellular ROS levels in bone marrow (BM) cells from AML-PDX samples were assessed after ex vivo exposure to brusatol, BSO, and doxorubicin. A DCF-based flow cytometry analysis showed markedly increased ROS levels in BM cells exposed to BSO and brusatol (Figure 6C). To determine if mitochondrial levels of ROS were increased by NRF2 targeting with anthracyclines, a MitoSOX-based flow cytometry analysis was adopted using ex vivo AML-PDX samples or MOLM parental or TKI-resistant bone marrow cells [33]. Figure 6D shows higher mitochondrial ROS formation as quantified by the mean fluorescence intensity (MFI) for MitoSOX in brusatol- and daunorubicin-treated cells compared to single-agent treatment (Figure 6D).

To determine if this combination was effective in vivo, we utilized an NSG-SGM3 based AML-PDX mouse model. Briefly, cells from samples AML-1 and AML-13 were introduced by tail vein injection. The engraftment efficiency/leukemic burden was measured in peripheral blood by cytometric analysis of human CD45^+^ cells, starting at seven days after xenotransplantation. When the leukemic burden was between 0.5 and 2%, treatment was started using brusatol and/or anthracyline (daunorubicin) either alone or in combination. As seen in Figure 6E, treatment with brusatol or daunorubicin alone had a moderate effect on the extended survival of the mice bearing disease. Interestingly, the combination of brusatol and daunorubicin was the most effective, resulting in a significant (*p* < 0.05) increase in the survival of the animals (Figure 6E). 

Next, we determined whether brusatol can potentiate anthracycline therapeutic affects in additional in vivo models. Four weeks after beginning of initial treatment with brusatol, daunorubicin, or the combination, the peripheral blood lymphocytes (PBL) and bone marrow aspirates of each experimental group were harvested, and engraftment efficiency was measured by flow cytometry analysis of hCD45^+^ LB. Brusatol-based treatment groups showed a greater reduction in human CD45^+^ cells in peripheral blood (Figure 6F) or bone marrow (Figure 6G) when compared to vehicle-treated groups. Furthermore, the combination treatment groups of brusatol and daunorubicin had significant potentiation of effects beyond single-agent treatment, although the 2 mg/kg dose of brusatol was uniformly quite effective at reducing leukemia burden. These data collectively show that combined treatment of brusatol and daunarubucin can reduce leukemia progression and prolong survival of mice bearing FLT3-ITD AML. 

## 4. Discussion

Our data are the first to highlight the activation of NRF2 leading to HO-1 expression as targetable features of FLT3 mutant AML. We demonstrate this by knockdown and pharmacological approaches. For the latter, the use of brusatol, a botanical natural product, in this subset of AML is novel and shows strong in vivo efficacy particularly when combined with anthracycline treatment. 

HO-1, also known as heat shock protein 32 (HSP32), has been functionally implicated in both chronic myeloid leukemia and AML, where it is capable of neutralizing free radical-producing heme [27]. However, HO-1 differs from other antioxidants in that it can also play a role in the immune response, survival and growth, apoptotic response, and metabolism [34]. Specifically in AML cells, HO-1 has been previously noted to promote resistance to apoptosis induced by TNFα [35], cytarabine, and daunorubicin [4]. Interestingly, HO-1 was noted to be up-regulated in patient samples from the French-American-British (FAB) M4 subdivision of AML (myelomonocytic) as compared to other categories [36]. This sub-classification is also enriched in FLT3-ITD expressing patients [37]. Furthering evidence that HO-1 may be important in the FLT3 mutant setting, our data show that patients with FLT3-ITD disease with elevated HO-1 expression are more likely to have inferior OS as compared to their low-expressing counterparts (Figure 1A). In our model cell lines, not only is HO-1 elevated at the mRNA level in ITD-expressing cells compared to wild-type (Figure 1B,C), but there is also increased HO-1 protein in ITD-positive cells that have resistance to targeted therapeutics (Figure 1D,E). In both TKI-sensitive and -resistant models, HO-1 indeed plays a functional role promoting proliferation (Figure 1F), since the knockdown of HO-1 results in decreased proliferation of both ITD and ITDR cells. Our data also highlight the potential for the use of targeting HO-1 in TKI-resistant disease as both knockdown and targeting of HO-1 with ZnPP result in increased sensitivity to quizartinib, a second-generation Flt3-directed TKI. 

Indirect targeting of HO-1 is also evaluated in our study since there are a number of transcription factors that are attributed to regulation of HO-1 [38]. Transcriptional activation of HO-1 by NRF2, a master regulator of antioxidant transcription, known to contribute to leukemogenesis, is highlighted in our study. Through binding to antioxidant response elements, NRF2 controls several antioxidant genes and bolsters the levels of thioredoxin, peroxiredoxin, and glutathione, as well as HO-1. NRF2 activity is elevated in some types of leukemia and can promote resistance to treatments such as proteasome inhibitors in AML [27]. We have found that NRF2 is expressed across FLT3 mutant cells (Figure 3), highlighting the potential requirement for targeting activity, not expression, of NRF2. Pharmacological inhibitors of NRF2 are an emerging area of drug development; however, here we focus on the quassinoid compound, brusatol, which has been evaluated in some AML models, and has been shown to sensitize to cytarabine. Specifically, brusatol causes a decrease in NRF2 protein resulting in increased sensitivity to chemotherapeutics or chemical stress [28,39,40]. Here, we extend this finding to FLT3-ITD models and show additional combination strategies. We confirm that brusatol decreased NRF2 protein (Figure 3) in human FLT3-ITD expressing cells, and a subsequent decrease in HO-1 protein levels, suggesting that HO-1 is a downstream target of NRF2 in ITD+ AML cells (Figure 3). Interestingly, both TKI-resistant and -sensitive cell lines and primary patient samples show similar responses to brusatol dosing (Figure 3). 

Over the last three decades, multiple clinical studies have targeted NRF2, but none have focused on FLT3 mutant AML [41,42], although the targeting of NRF2 and antioxidants has been implicated in leukemic stem cell (LSC) depletion and has shown potential for further study [43,44]. A need for biomarkers of NRF2 inhibition has been articulated across cancer types, and our data suggest that HO-1 or downstream redox changes such as ROS levels or GSH/GSSG ratios may serve this purpose in AML. Our in vivo data shown in Figure 5 and Figure 6 highlight the antileukemic effects of the co-treatment of brusatol and anthracyclines, prolonging the survival of AML-bearing mice and efficiently suppressing leukemic burden in the blood as well as in the bone marrow. The effects of these combinations have potential for enhancing clinical treatment of ITD-positive patients with or without resistance to TKI. 

## 5. Conclusions

Our findings suggest that targeting HO-1 or NRF2 in FLT3-ITD-bearing AML may augment responses to therapeutics.

## Data Availability

All of the data is contained within the article.

## References

[1] Molica M., Breccia M., Foa R., Jabbour E., Kadia T.M. (2019). Maintenance therapy in AML: The past, the present and the future. Am. J. Hematol..

[2] Kantarjian H., Kadia T., DiNardo C., Daver N., Borthakur G., Jabbour E., Garcia-Manero G., Konopleva M., Ravandi F. (2021). Acute myeloid leukemia: Current progress and future directions. Blood Cancer J..

[3] Kennedy V.E., Smith C.C. (2020). FLT3 Mutations in Acute Myeloid Leukemia: Key Concepts and Emerging Controversies. Front. Oncol..

[4] Daver N., Schlenk R.F., Russell N.H., Levis M.J. (2019). Targeting FLT3 mutations in AML: Review of current knowledge and evidence. Leukemia.

[5] Chillon M.C., Fernandez C., Garcia-Sanz R., Balanzategui A., Ramos F., Fernandez-Calvo J., Gonzalez M., Miguel J.F. (2004). FLT3-activating mutations are associated with poor prognostic features in AML at diagnosis but they are not an independent prognostic factor. Hematol. J..

[6] Gunawardane R.N., Nepomuceno R.R., Rooks A.M., Hunt J.P., Ricono J.M., Belli B., Armstrong R.C. (2013). Transient exposure to quizartinib mediates sustained inhibition of FLT3 signaling while specifically inducing apoptosis in FLT3-activated leukemia cells. Mol. Cancer Ther..

[7] Cortes J.E., Khaled S., Martinelli G., Perl A.E., Ganguly S., Russell N., Kramer A., Dombret H., Hogge D., Jonas B.A. (2019). Quizartinib versus salvage chemotherapy in relapsed or refractory FLT3-ITD acute myeloid leukaemia (QuANTUM-R): A multicentre, randomised, controlled, open-label, phase 3 trial. Lancet Oncol..

[8] Antar A.I., Otrock Z.K., Jabbour E., Mohty M., Bazarbachi A. (2020). FLT3 inhibitors in acute myeloid leukemia: Ten frequently asked questions. Leukemia.

[9] Perl A.E., Martinelli G., Cortes J.E., Neubauer A., Berman E., Paolini S., Montesinos P., Baer M.R., Larson R.A., Ustun C. (2019). Gilteritinib or Chemotherapy for Relapsed or Refractory FLT3-Mutated AML. N. Engl. J. Med..

[10] Sillar J.R., Germon Z.P., DeIuliis G.N., Dun M.D. (2019). The Role of Reactive Oxygen Species in Acute Myeloid Leukaemia. Int. J. Mol. Sci..

[11] Jayavelu A.K., Moloney J.N., Bohmer F.D., Cotter T.G. (2016). NOX-driven ROS formation in cell transformation of FLT3-ITD-positive AML. Exp. Hematol..

[12] Rushworth S.A., Zaitseva L., Langa S., Bowles K.M., MacEwan D.J. (2010). FLIP regulation of HO-1 and TNF signalling in human acute myeloid leukemia provides a unique secondary anti-apoptotic mechanism. Oncotarget.

[13] Furfaro A.L., Traverso N., Domenicotti C., Piras S., Moretta L., Marinari U.M., Pronzato M.A., Nitti M. (2016). The Nrf2/HO-1 Axis in Cancer Cell Growth and Chemoresistance. Oxid. Med. Cell. Longev..

[14] Wang T., Dou Y., Lin G., Li Q., Nie J., Chen B., Xie J., Su Z., Zeng H., Chen J. (2021). The anti-hepatocellular carcinoma effect of Brucea javanica oil in ascitic tumor-bearing mice: The detection of brusatol and its role. Biomed. Pharmacother..

[15] Sankaranarayanan K., Jaiswal A.K. (2004). Nrf3 negatively regulates antioxidant-response element-mediated expression and antioxidant induction of NAD(P)H:quinone oxidoreductase1 gene. J. Biol. Chem..

[16] Kannan S., Muthusamy V.R., Whitehead K.J., Wang L., Gomes A.V., Litwin S.E., Kensler T.W., Abel E.D., Hoidal J.R., Rajasekaran N.S. (2013). Nrf2 deficiency prevents reductive stress-induced hypertrophic cardiomyopathy. Cardiovasc. Res..

[17] Kannan S., Aitken M.J.L., Herbrich S.M., Golfman L.S., Hall M.G., Mak D.H., Burks J.K., Song G., Konopleva M., Mullighan C.G. (2019). Antileukemia Effects of Notch-Mediated Inhibition of Oncogenic PLK1 in B-Cell Acute Lymphoblastic Leukemia. Mol. Cancer Ther..

[18] Armstrong J.S., Steinauer K.K., Hornung B., Irish J., Lecane P., Birrell G., Peehl D.M., Knox S.J. (2002). Role of glutathione depletion and reactive oxygen species generation in apoptotic signaling in a human B lymphoma cell line. Cell Death Differ..

[19] Kannan S., Sutphin R.M., Hall M.G., Golfman L.S., Fang W., Nolo R.M., Akers L.J., Hammitt R.A., McMurray J.S., Kornblau S.M. (2013). Notch activation inhibits AML growth and survival: A potential therapeutic approach. J. Exp. Med..

[20] Irwin M.E., Rivera-Del Valle N., Chandra J. (2013). Redox control of leukemia: From molecular mechanisms to therapeutic opportunities. Antioxid. Redox Signal..

[21] Podkalicka P., Mucha O., Jozkowicz A., Dulak J., Loboda A. (2018). Heme oxygenase inhibition in cancers: Possible tools and targets. Contemp. Oncol..

[22] Dany M., Gencer S., Nganga R., Thomas R.J., Oleinik N., Baron K.D., Szulc Z.M., Ruvolo P., Kornblau S., Andreeff M. (2016). Targeting FLT3-ITD signaling mediates ceramide-dependent mitophagy and attenuates drug resistance in AML. Blood.

[23] He Z., Zhang S., Ma D., Fang Q., Yang L., Shen S., Chen Y., Ren L., Wang J. (2019). HO-1 promotes resistance to an EZH2 inhibitor through the pRB-E2F pathway: Correlation with the progression of myelodysplastic syndrome into acute myeloid leukemia. J. Transl. Med..

[24] Konturek-Ciesla A., Radziszewska A., Ciesla M., Czmoczek A., Zukowska M., Dulak J., Jozkowicz A., Bukowska-Strakova K. (2018). Abstract 2824: Charting the landscape of genomic instability in acute myeloid leukemia: Interaction between G-quadruplexes and heme oxygenase-1 in leukemia. Cancer Res..

[25] Chen Y., Pan Y., Guo Y., Zhao W., Ho W.T., Wang J., Xu M., Yang F.C., Zhao Z.J. (2017). Tyrosine kinase inhibitors targeting FLT3 in the treatment of acute myeloid leukemia. Stem Cell Investig..

[26] Hirai K., Sasahira T., Ohmori H., Fujii K., Kuniyasu H. (2007). Inhibition of heme oxygenase-1 by zinc protoporphyrin IX reduces tumor growth of LL/2 lung cancer in C57BL mice. Int. J. Cancer.

[27] Irwin M.E., Johnson B.P., Manshouri R., Amin H.M., Chandra J. (2015). A NOX2/Egr-1/Fyn pathway delineates new targets for TKI-resistant malignancies. Oncotarget.

[28] Olayanju A., Copple I.M., Bryan H.K., Edge G.T., Sison R.L., Wong M.W., Lai Z.Q., Lin Z.X., Dunn K., Sanderson C.M. (2015). Brusatol provokes a rapid and transient inhibition of Nrf2 signaling and sensitizes mammalian cells to chemical toxicity-implications for therapeutic targeting of Nrf2. Free Radic. Biol. Med..

[29] Thimmulappa R.K., Lee H., Rangasamy T., Reddy S.P., Yamamoto M., Kensler T.W., Biswal S. (2006). Nrf2 is a critical regulator of the innate immune response and survival during experimental sepsis. J. Clin. Investig..

[30] Xiao W., Loscalzo J. (2020). Metabolic Responses to Reductive Stress. Antioxid. Redox Signal..

[31] Tabe Y., Saitoh K., Yang H., Sekihara K., Yamatani K., Ruvolo V., Taka H., Kaga N., Kikkawa M., Arai H. (2018). Inhibition of FAO in AML co-cultured with BM adipocytes: Mechanisms of survival and chemosensitization to cytarabine. Sci. Rep..

[32] Traverso N., Ricciarelli R., Nitti M., Marengo B., Furfaro A.L., Pronzato M.A., Marinari U.M., Domenicotti C. (2013). Role of glutathione in cancer progression and chemoresistance. Oxid. Med. Cell. Longev..

[33] Kauffman M.E., Kauffman M.K., Traore K., Zhu H., Trush M.A., Jia Z., Li Y.R. (2016). MitoSOX-Based Flow Cytometry for Detecting Mitochondrial ROS. React. Oxyg. Species.

[34] Loboda A., Jozkowicz A., Dulak J. (2015). HO-1/CO system in tumor growth, angiogenesis and metabolism—Targeting HO-1 as an anti-tumor therapy. Vasc. Pharmacol..

[35] Shirley S., Micheau O. (2010). The heme oxygenase-1 and c-FLIP in acute myeloid leukemias: Two non-redundant but mutually exclusive cellular safeguards protecting cells against TNF-induced cell death?. Oncotarget.

[36] Yong S.B., Chung J.Y., Kim S.S., Choi H.S., Kim Y.H. (2020). CD64-targeted HO-1 RNA interference enhances chemosensitivity in orthotopic model of acute myeloid leukemia and patient-derived bone marrow cells. Biomaterials.

[37] Kuchenbauer F., Kern W., Schoch C., Kohlmann A., Hiddemann W., Haferlach T., Schnittger S. (2005). Detailed analysis of FLT3 expression levels in acute myeloid leukemia. Haematologica.

[38] Durante W. (2010). Targeting heme oxygenase-1 in vascular disease. Curr. Drug Targets.

[39] Ren D., Villeneuve N.F., Jiang T., Wu T., Lau A., Toppin H.A., Zhang D.D. (2011). Brusatol enhances the efficacy of chemotherapy by inhibiting the Nrf2-mediated defense mechanism. Proc. Natl. Acad. Sci. USA.

[40] Rajagopal C., Lankadasari M.B., Aranjani J.M., Harikumar K.B. (2018). Targeting oncogenic transcription factors by polyphenols: A novel approach for cancer therapy. Pharmacol. Res..

[41] Yagishita Y., Gatbonton-Schwager T.N., McCallum M.L., Kensler T.W. (2020). Current Landscape of NRF2 Biomarkers in Clinical Trials. Antioxidants.

[42] Zhang D., Hou Z., Aldrich K.E., Lockwood L., Odom A.L., Liby K.T. (2021). A Novel Nrf2 Pathway Inhibitor Sensitizes Keap1-Mutant Lung Cancer Cells to Chemotherapy. Mol. Cancer Ther..

[43] Pearson K.J., Lewis K.N., Price N.L., Chang J.W., Perez E., Cascajo M.V., Tamashiro K.L., Poosala S., Csiszar A., Ungvari Z. (2008). Nrf2 mediates cancer protection but not prolongevity induced by caloric restriction. Proc. Natl. Acad. Sci. USA.

[44] Jones C.L., Stevens B.M., D’Alessandro A., Culp-Hill R., Reisz J.A., Pei S., Gustafson A., Khan N., DeGregori J., Pollyea D.A. (2019). Cysteine depletion targets leukemia stem cells through inhibition of electron transport complex II. Blood.

